# Ebola hemorrhagic fever: current outbreak and progress in finding a cure

**DOI:** 10.1186/s40199-014-0070-9

**Published:** 2014-11-12

**Authors:** Soodabeh Saeidnia, Mohammad Abdollahi

**Affiliations:** Medicinal Plants Research Center, Faculty of Pharmacy, Tehran University of Medical Sciences, P. O. Box 14155–6451, Tehran, Iran; Faculty of Pharmacy, and Pharmaceutical Sciences Research Center, Tehran University of Medical Sciences, Tehran, 1417614411 Iran

## Introduction

As reported: “A health care worker at Texas Health Presbyterian Hospital, who cared for dying Ebola patient has been tested positive for the virus after a preliminary test, officials said early Sunday. If confirmed, it would be the first known person-to-person transmission of the disease in the United States” [1].

Every day people hear bad news around the distribution of Ebola in Africa and also the possibility of its changing to other continents. Coming up a successful remedy for this horrible disease of the century is expected but inventing and producing adequate medicines to be usable for the increasing number of patients may have challenges. The only available drug “Zmapp” has been produced by the biotech firm “Mapp Biopharmaceutical Inc.”, which is based in San Diego. “Mapp Biopharmaceutical” is working with the “National Institutes of Health and the Defense Threat Reduction Agency” that has followed to discover and develop an Ebola medicine for several years [2]. Nevertheless, scientists think that casting out an untested drug during an enormous outbreak would be really hard. Furthermore, “Zmapp” is only a medicine under evaluation and typically made in low amounts. On the other hand, clinical trial and tracking the success of such drugs requires more human resources and volunteers for an excursion to the affected regions, whereas the number of professional and medical staffs in those countries is scarce and inadequate.

Despite the enormous attraction of research on the Ebola drug discovery and development, running on this virus is not simply accessed, because it requires biosafety level 4-equivalent containment. Unfortunately, despite high standards of security in such labs, research laboratory workers are still at risk of contracting Ebola hemorrhagic fever especially during animal experimentation. Until 2011, three laboratory accidents with Ebola virus were documented in the literature and one of them led to mortality [3]. That experimental medicine has been so far administered to two American patients [4].

As of 1976 (the date of Ebola emerged), Ebola hemorrhagic fever affected poor countries and such a neglected diseases have not been a research priority for pharmaceutical companies [4] but now it seems a real research priority.

### Chronological history

Undoubtedly, Ebola epidemic in 2014 is the biggest epidemic of this virus, so far, since multiple countries in the West-Africa have been feigned. Established on the present information, a few events have been reported in Nigeria and a single case reported in Senegal but fortunately not further spread in those lands. Nevertheless, the first “travel-associated” case of Ebola was diagnosed in the United States on September 30, 2014. As of the date of writing this article, it is described that the US Center for Disease Control and Prevention (CDC) and collaborators are taking precautions to prevent the spread of Ebola within the US in association with other governmental agencies, the World Health Organization (WHO), as well as other domestic and international collaborators. Furthermore, CDC has outspread teams of health care professionals to West-Africa and other affected countries [5].

Historically for the first time, Ebola (referred to the figure of “Ebola River” in Zaire) was struck in Sudan and Zaire (1976). Bibliography shows that the first outbreak of Ebola (Ebola-Sudan) infected over 284 people (mortality rate: 53%), while the second Ebola virus appeared in Yambuku, Zaire, Ebola-Zaire (EBOZ) some months later with the highest mortality rate (88%), infected 318 people. Notwithstanding the great efforts of dedicated researchers so far, its natural reservoir has never been set up. The third form of Ebola (Ebola Reston, EBOR), was found in 1989 in infected monkeys that were imported into Virginia from the Philippines. All the same, a few individuals infected and never developed Ebola hemorrhagic fever (EHF). The last known strain of Ebola (Ebola Cote d’Ivoire, EBO-CI) was emerged in 1994 during a necropsy on a dead chimpanzee from the Tai Forest, Cote d’Ivoire. The etiologist who did necropsy accidentally infected herself [6].

An overview of chronology of Ebola, published by US CDC [5], expressed that the recent outbreak of Ebola (2014) is the biggest epidemic in history because of the multi-countries involved and tremendous number of diagnosed patients (4655 people reported until the date of this article) that unfortunately spread more.

### Reservoirs of Ebola

Bats are known the most probably natural reservoirs of Ebola virus, followed by plants, arthropods, and birds. In the first epidemic, bats were found in the cotton manufactory, in which the first cases of infection were observed (1976, 1979). In reality, bats are the only infected animals with no clinical signs among 24 plants and 19 vertebrate species that experimentally inoculated with Ebola virus [7,8]. Moreover, fruit bats are used by people in some fields of West-Africa as smoked and grilled or even utilized in preparation of a spicy soup. It is described that although transmission between natural sources and humans rarely occur, outbreaks are mostly originated from an individual who handles the carcass of gorilla, chimpanzee or Duiker [9,10].

### Virology of Ebola

The Ebola virus was thought to be a new strain of the Marburg virus, but it was renamed “Ebola virus” in 2010 referred to the name of “Ebola River” in its original country, Congo (formerly named Zaire). In fact, this virus is just a single member of the species “Zaire Ebolavirus” in the genus Ebolavirus, family Filoviridae. The Zaire species of the Ebola virus are the most dangerous, causing almost 90% mortality rate [11]. The genome of Ebola virus is a single-stranded RNA, including about 19,000 nucleotides [12]. Ebolavirions are filamentous particles that can be observed in a shepherd’s crook or “U” or a “6” features, and also they may be coiled, toroid, or branched. In addition, Ebolavirions are 80 nm in width, while their length is somewhat variable [13,14].

### Pathophysiology of Ebola hemorrhagic fever

Literature reveals filoviruses have a broad spectrum cells to target in susceptible host species, of which monocytes, macrophages, dendritic cells (DCs), hepatocytes, adrenal cortical cells, fibroblasts and endothelial cells. The replication of virus is centered around the cells of the mononuclear phagocyte system resulting in quick circulation and widespread transferring throughout the host body [15]. The Ebola virus glycoprotein (GP) is synthesized in a secreted (sGP) or full-length transmembrane form, each of which well-defines its biochemical and biological properties. GP is known to form a trimeric complex (30) and binds to endothelial cells, while sGP does not bind to epithelial cells. It forms a dimeric protein that interferes with the signaling of neutrophils, and therefore enables the virus to put off the immune system through suppressing the early steps of neutrophil activation. Infected neutrophils also act as carriers to transport the virus throughout the body to locate like the lymph nodes, liver, lungs, and spleen [15,16].

As literature shows, filoviruses are able to enter the target cells through several uptake mechanisms such as lipid raft-dependent and receptor-mediated endocytosis, as well as macropinocytosis. When viruses fuse into cellular membrane, nucleocapsids are released into the cytoplasm and play a role of template for transcription and replication. Concisely, the replicated RNAs are encapsidated by the nucleocapsid proteins while nucleocapsids are transported to the locations of viral release, where budding is ongoing. Viral assembling and budding might happen either at intracellular membranes (multivesicular budies) or at the plasma membrane [15,17]. As a matter of fact, direct infection of monocytes and macrophages results in releasing cytokines related to inflammation and fever because of the host immune responses to Ebola virus and cell damage. On the other side, infected endothelial cells are able to induce a cytopathic damage to the endothelial barrier that contributes to the loss of vascular integrity, especially alongside the cytokine effects [18].

### Cell destination

**Figure 1:**
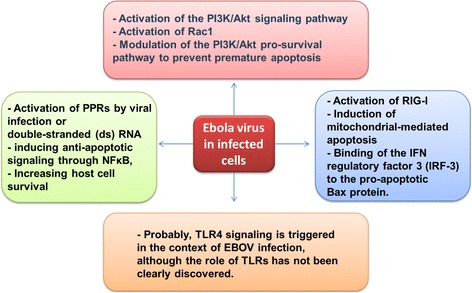
**Interactions of Ebola viruses with cell signaling pathways; EBOV: Ebola Virus; IFN: Interferons; NFkB: Nuclear factor NF-kappa-B; PI3K/Akt: Phosphatidylinositol-3-Kinase and Protein Kinase B; PPRs: Peroxisome Proliferator-activated Receptors; RIG-1: Retinoic acid-Inducible Gene 1; TLR4: Toll-Like Receptor 4.**

Investigations of filovirus showed that they exploit the infected cells to replicate successfully that potentially may lead to cell destruction. Intriguingly, viruses exhibited mechanisms to avoid cell death, because destroying the infected cells inhibits the production of progeny viruses. The mentioned mechanisms have been developed through a complicated interaction between viruses and cell death signaling pathways [19]. However, electron microscopic studies and biochemical analyses of tissues isolated from infected animals exhibited that infected macrophages, dendritic cells, hepatocytes, and endothelial cells did not develop apoptosis, since the cells looked normal in morphological studies, whereas necrosis was noted in some shells. These findings reveal that filoviruses are able to control the cell death signaling and pathways. For example, in Ebola infected cells, the energizing of the PI3K/Akt signaling pathway can take place very early leading to activation of Rac1, which is a regulator of endocytosis and vesicular trafficking [15,20]. Filoviruses are known to interfere with antiviral signaling pathways. The schematic diagram (Figure 1) summarized some of these interventions.

There is evidence that Ebola virus surface protein (GP) plays a role in cytotoxicity and cell damage, inducing cell rounding and detachment as well as membrane permeabilization. Furthermore, GP expression can down-modulate the cell surface proteins such as adhesion molecules, MHC class I proteins, and EGF receptor. Literature demonstrated that the GP-induced cytotoxicity may happen due to interaction of GP with the GTPase dynamin, resulting in noise with the intracellular trafficking of cell surface proteins [21]. On the other side, lymphocytes (CD4 and CD8 T cells and Natural Killer cells) do not support virus replication, likely due to the lack of receptors, nevertheless they can develop apoptosis while they are not tainted. A 17-mer in filovirus GPs has recently been reported to induce lymphocyte death and suppression of cytokine responses. This 17-mer is responsible for resembling an immunosuppressive motive. But the doubt, nevertheless remains that how it is possible, since lymphocytes do not attach to the viral GP? [22].

### Prevention strategies

It is easily-documented that small interfering RNAs (siRNAs) and phosphorodiamidate morpholino oligomers (PMOs) could target Ebola virus RNA polymerase L protein to prevent disease in nonhuman primates [23,24]. These are some cases of advanced antisense approaches for therapy of Ebola virus. Moreover, TKM-Ebola and Sarepta Therapeutics are now being tested in the Phase I clinical trial in humans [25]. ZMapp that has been successfully used for two American patients is a monoclonal antibody vaccine. As it was already reported, the total supply of this medication is restricted and should be under more investigations to gain significant results.

### Treatment

Although there is no specific approved medicine, early supportive care, rehydration and symptomatic cures such as using RBC, platelets or fresh frozen plasma, as well as heparin to prevent disseminated intravascular coagulation, and clotting agents to decrease bleeding can be helpful [26]. Maintaining blood volume and electrolyte balance as well as treating other bacterial infections and dialysis may be employed [27].

Presently, there is no ground for using alternative therapies or traditional medicine for prevention or treatment of Ebola hemorrhagic fever. US FDA advises people to be careful of advertisements for the benefits of different anti-Ebola products [28]. Nevertheless, some of antiviral drugs are now being considered. Among them, favipiravir was already approved in Japan against influenza pandemics. Literature revealed that this compound can be efficient *in vitro* on Ebola infected mice. The most recent news show that this drug has been successful in the treatment of a French woman got Ebola in Liberia. The woman received three experimental drugs for the treatment that one of them was favipiravir [29,30].

BCX4430 (a broad-spectrum small molecule by BioCryst Pharmaceuticals), brincidofovir (another broad-spectrum antiviral granted an emergency FDA approval) and lamivudine (an antiviral drug used to treat HIV/AIDS) have recently been reported to show positive results in treatment of Ebola-infected patients [31]. In fact, due to the lack of available treatment, a number of possible antiviral candidates that targeted against Ebola, including both natural products (such as scytovirin and griffithsin) and synthetic drugs (like FGI-103, FGI-104, FGI-106, dUY11 and LJ-001) are presently under investigation [32-34].

**Figure 2:**
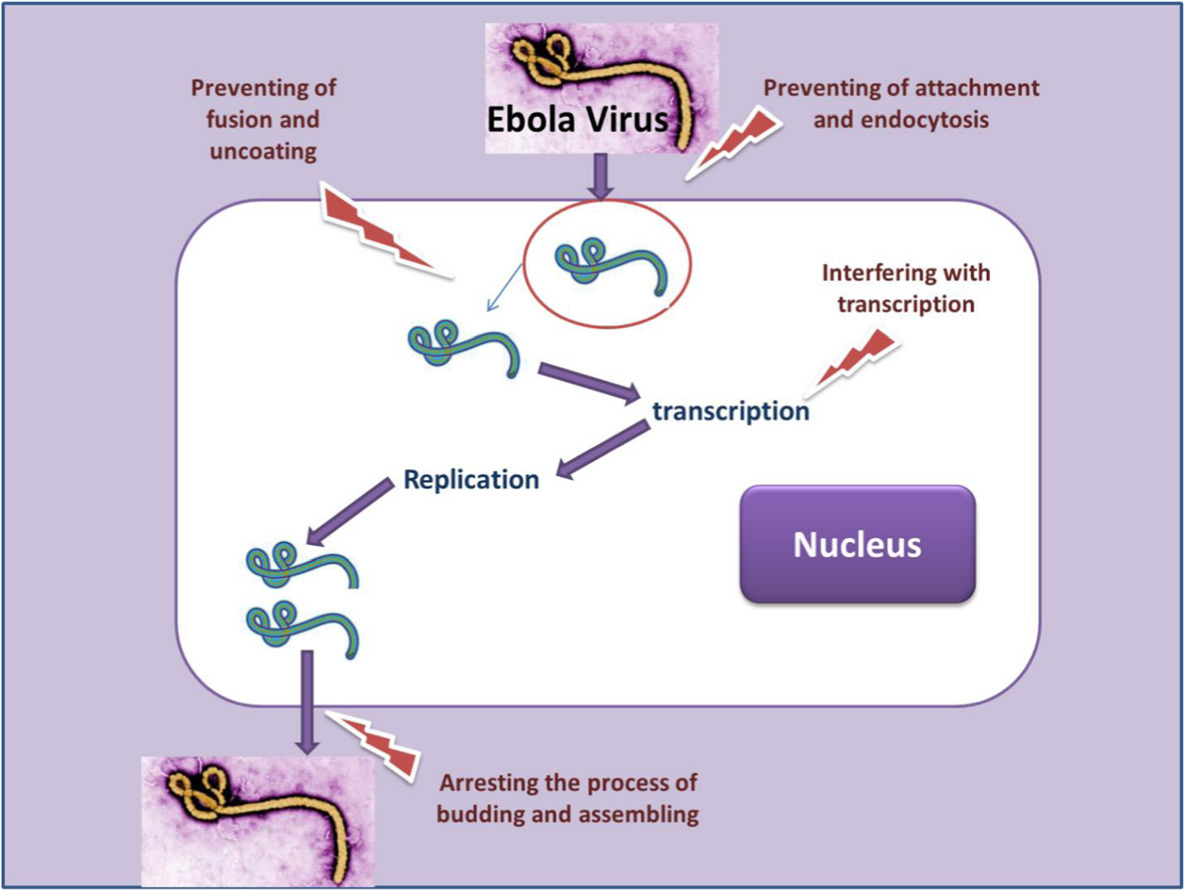
**Possible sites of actions and targets for anti-Ebola drug discovery.**

Alongside other antiviral drugs that are under investigations, scientists could find a number of *in vitro*-effective known drugs that are currently being used for other indications including ion channel blockers (for treatment of heart arrhythmias) like amiodarone, dronedarone and verapamil, which could block up the entry of Ebola virus into cells [35]. In sum, the well-known selective estrogen receptor modulators (clomiphene and toremifene) that are being employed in therapy of infertility and breast cancer, have been reported to suppress the progress of Ebola virus both *in vitro* and *in vivo* [36]. Since these drugs are being used orally and historically for other human illnesses, they can be candidates for treatment of Ebola hemorrhagic fever in the regions that are very far away alone or alongside other antiviral medicines. Figure 2 shows the possible targets for the future anti-Ebola drugs.

It seems that patients, who have rescued from Ebola after infection, should possess strong immunity system that acted faster than Ebola virus and restricted its destroying activity. Therefore, synthetic or naturally immunostimulats may help patients in combination therapy with anti-Ebola medicines. Furthermore as mentioned above, if estrogen receptor modulators (like clomiphene) would probably be effective in clinical trials, other phyto-estrogens from different classes of secondary metabolites like isoflavonoids and phytosterls could be candidates for *in vitro* and *in vivo* experiments. Successfulness of natural proteins like “Scytovirin” that is a cyanobacterial based (95-amino acid) antiviral candidate leads scientists to another group of compounds which could be produced through biotechnological approaches.

## Conclusion

Although there is some information about the Ebola virus and its interaction with infected or non-infected cells, its target cells, stages of the replication cycle, and its cytopathic effects, until now we are far from having an efficient medication to control the virus outbreak. To be prosperous in the founding of a medicine for Ebola, the signaling pathways in the infected cells that contribute in replication and fate must be better elucidated. There are promising candidates in clinical trials for prevention of the disease like DNA vaccines or vaccines derived from adenoviruses, vesicular stomatitis Indiana virus (VSIV) or filovirus-like particles (VLPs). These vaccines could protect nonhuman primates from Ebola and hopefully can be engaged in human [37-40].
